# Dieth­yl(hy­droxy)ammonium 3-carb­oxy­benzoate

**DOI:** 10.1107/S1600536810030746

**Published:** 2010-08-18

**Authors:** Yun Guo, Cong Wang, Hai-Xiang Chen

**Affiliations:** a College of Pharmaceutical Sciences, Zhejiang University of Technology, Hangzhou 310014, People’s Republic of China; b Analytical Center, Zhejiang Sci-Tech University, Hangzhou 310018, People’s Republic of China

## Abstract

In the title molecular compound, C_4_H_12_NO^+^·C_8_H_5_O_4_^−^, the *N*,*N*-dieth­yl(hy­droxy)ammonium cation (DTHA) is linked to the 3-carb­oxy­benzoate anion (HBDL) by O—H⋯O and N—H⋯O hydrogen bonds with a graph-set motif  *R*_2_^2^(7). In the crystal, helical chains are formed by O—H⋯O hydrogen bonds, propagating along [010]. The crystal structure is further stabilized by π–π inter­actions between inversion-related HBDL benzene rings [centroid–centroid distance = 3.900 (4) Å] and C—H⋯O inter­actions.

## Related literature

For supra­molecular structures comprising benzene-dicarb­oxy­lic acids, see: Karpova *et al.* (2004 ▶); Bourne *et al.* (2001 ▶); Jin *et al.* (2005 ▶); Dale *et al.* (2004 ▶); Shan *et al.* (2002 ▶); Yuge *et al.* (2006 ▶); Zhao *et al.* (2007 ▶). For graph-set analysis, see: Etter (1990 ▶); Bernstein *et al.* (1995 ▶).
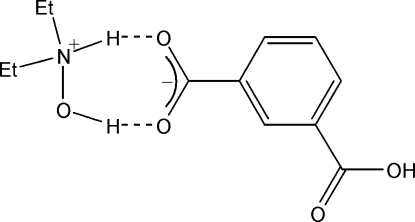


## Experimental

### 

#### Crystal data

C_4_H_12_NO^+^·C_8_H_5_O_4_^−^
*M* *_r_* = 255.27
Monoclinic, 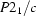

*a* = 9.535 (7) Å
*b* = 11.937 (9) Å
*c* = 11.660 (9) Å
β = 96.959 (15)°
*V* = 1317.4 (17) Å^3^
*Z* = 4
Mo *K*α radiation
μ = 0.10 mm^−1^
*T* = 273 K
0.34 × 0.15 × 0.14 mm

#### Data collection

Bruker SMART CCD area-detector diffractometer
Absorption correction: multi-scan (*SADABS*; Bruker, 2001 ▶) *T*_min_ = 0.967, *T*_max_ = 0.977
6749 measured reflections
2317 independent reflections
1912 reflections with *I* > 2σ(*I*)
*R*_int_ = 0.029

#### Refinement

*R*[*F* ^2^ > 2σ(*F* ^2^)] = 0.075
*wR*(*F* ^2^) = 0.203
*S* = 1.19
2317 reflections
167 parameters
H-atom parameters constrained
Δρ_max_ = 0.20 e Å^−3^
Δρ_min_ = −0.26 e Å^−3^

### 

Data collection: *SMART* (Bruker, 2007 ▶); cell refinement: *SAINT* (Bruker, 2007 ▶); data reduction: *SAINT*; program(s) used to solve structure: *SHELXS97* (Sheldrick, 2008 ▶); program(s) used to refine structure: *SHELXL97* (Sheldrick, 2008 ▶); molecular graphics: *SHELXTL* (Bruker, 2007 ▶); software used to prepare material for publication: *SHELXL97*.

## Supplementary Material

Crystal structure: contains datablocks global, I. DOI: 10.1107/S1600536810030746/su2200sup1.cif

Structure factors: contains datablocks I. DOI: 10.1107/S1600536810030746/su2200Isup2.hkl

Additional supplementary materials:  crystallographic information; 3D view; checkCIF report

## Figures and Tables

**Table 1:** Hydrogen-bond geometry (Å, °)

*D*—H⋯*A*	*D*—H	H⋯*A*	*D*⋯*A*	*D*—H⋯*A*
N1—H1⋯O1	0.91	1.85	2.702 (4)	155
O5—H5⋯O2	0.82	1.74	2.545 (4)	169
O4—H4⋯O1^i^	0.82	1.79	2.606 (4)	175
C10—H10*A*⋯O2^ii^	0.97	2.58	3.373 (5)	139
C12—H12*A*⋯O5^iii^	0.97	2.51	3.426 (5)	157
C12—H12*B*⋯O3^iv^	0.97	2.53	3.117 (5)	119

Symmetry codes: (i) 
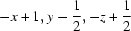
; (ii) 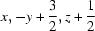; (iii) 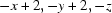; (iv) 
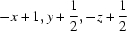
.

**Fig. 1.**
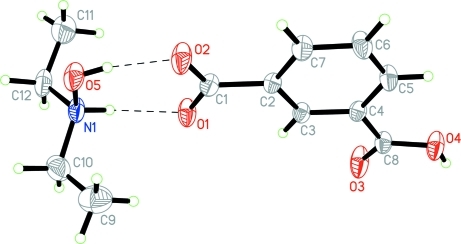
The molecular structure, and atom-numbering scheme, of the title compound. The displacement ellipsoids have been drawn at the 50% probability level. One DTHA cation and one HBDL anion are linked via O—H···O and N—H···O hydrogen bonds (dashed lines); see Table 1 for details.

**Fig. 2.**
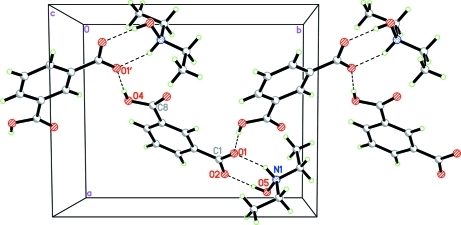
A view along the c-axis of the crystal packing of the title compound. The intermolecular O-H···O amd N-H···O hydrogen bonds are shown by dashed lines [symmetry operation: (') = 1-x, -1-y, -z].

## supplementary crystallographic information

### Comment

Various supramolecular structures comprising benzene-dicarboxylic acids have been reported (Bourne *et al.*, 2001; Shan *et al.*, 2002; Karpova *et al.*, 2004; Dale *et al.*, 2004; Jin *et al.*, 2005; Yuge *et al.*, 2006; Zhao *et al.*, 2007). Continuing our research on such compounds, the title compound was synthesized and its crystal structure is described herein.

As shown in Fig. 1, the *N*,*N*-diethylhydroxylammonium cation (DTHA) is linked to the 3-carboxybenzoate anion (HBDL) by N1—H1···O1 and O5—H5···O2 hydrogen bonds (Table 1), which can be described in graph-set terminology as *R*^2^_2_(7) (Bernstein *et al.*, 1995). In the HBDL anion the COO^-^ group is only slightly inclined to the phenyl ring, by 4.4 (4)/%. In contrast the dihedral angle between the phenyl ring and the COOH group is 11.8 (4)/%.

In the crystal molecules are linked by O4—H4···O1^i^ (symmetry code: (i) 1-*x*, -1-*y*, -*z*) hydrogen bonds to form helical chains propagating in [010] (Table 1, Fig. 2). The hydrogen bond pattern can be described in graph-set terminology as C^1^_1_(8)*R*^2^_2_(7). The molecules are further associated by π–π interactions, involving the HBDL benzene rings related by an inversion center, with a centroid-to-centroid distance of 3.900 (4) Å. The structure is further stabilized by C-H···O contacts (Table 1).

### Experimental

*N*,*N*-diethylhydroxylammine and benzene-1,3-dicarboxylic acid, in a molar ratio of 1:1, were mixed and dissolved in sufficient ethanol by heating to 373 K, at which point a clear solution resulted. The reaction mixture was then cooled slowly to room temperature. Crystals of the title compound were formed, collected and washed with ethanol.

### Refinement

All the H-atoms were included in calculate positions and treated as riding atoms: O-H = 0.82 Å, N-H = 0.91 Å, and C-H = 0.93, 0.96 and 0.97 Å, for CH(aromatic), CH(methyl) and CH(methylene), respectively. *U*_iso_(H) = k × *U*_eq_ of the parent atom, where k = 1.5 for OH and methyl H-atoms and k = 1.2 for all other H-atoms.

### Crystal data

**Table:**

C_4_H_12_NO^+^·C_8_H_5_O_4_^−^	*F*(000) = 544
*M**_r_* = 255.27	*D*_x_ = 1.287 Mg m^−^^3^
Monoclinic, *P*2_1_/*c*	Mo *K*α radiation, λ = 0.71073 Å
Hall symbol: -P 2ybc	Cell parameters from 3574 reflections
*a* = 9.535 (7) Å	θ = 2.1–25.0°
*b* = 11.937 (9) Å	µ = 0.10 mm^−^^1^
*c* = 11.660 (9) Å	*T* = 273 K
β = 96.959 (15)°	Block, colorless
*V* = 1317.4 (17) Å^3^	0.34 × 0.15 × 0.14 mm
*Z* = 4	

### Data collection

**Table:**

Bruker SMART CCD area-detector diffractometer	2317 independent reflections
Radiation source: fine-focus sealed tube	1912 reflections with *I* > 2σ(*I*)
graphite	*R*_int_ = 0.029
phi and ω scans	θ_max_ = 25.0°, θ_min_ = 2.2°
Absorption correction: multi-scan (*SADABS*; Bruker, 2001)	*h* = −11→11
*T*_min_ = 0.967, *T*_max_ = 0.977	*k* = −14→13
6749 measured reflections	*l* = −13→9

### Refinement

**Table:**

Refinement on *F*^2^	Primary atom site location: structure-invariant direct methods
Least-squares matrix: full	Secondary atom site location: difference Fourier map
*R*[*F*^2^ > 2σ(*F*^2^)] = 0.075	Hydrogen site location: inferred from neighbouring sites
*wR*(*F*^2^) = 0.203	H-atom parameters constrained
*S* = 1.19	*w* = 1/[σ^2^(*F*_o_^2^) + (0.0962*P*)^2^ + 0.4181*P*] where *P* = (*F*_o_^2^ + 2*F*_c_^2^)/3
2317 reflections	(Δ/σ)_max_ < 0.001
167 parameters	Δρ_max_ = 0.20 e Å^−^^3^
0 restraints	Δρ_min_ = −0.26 e Å^−^^3^

### Special details

**Table:**

Geometry. All e.s.d.'s (except the e.s.d. in the dihedral angle between two l.s. planes) are estimated using the full covariance matrix. The cell e.s.d.'s are taken into account individually in the estimation of e.s.d.'s in distances, angles and torsion angles; correlations between e.s.d.'s in cell parameters are only used when they are defined by crystal symmetry. An approximate (isotropic) treatment of cell e.s.d.'s is used for estimating e.s.d.'s involving l.s. planes.
Refinement. Refinement of *F*^2^ against ALL reflections. The weighted *R*-factor *wR* and goodness of fit *S* are based on *F*^2^, conventional *R*-factors *R* are based on *F*, with *F* set to zero for negative *F*^2^. The threshold expression of *F*^2^ > σ(*F*^2^) is used only for calculating *R*-factors(gt) *etc*. and is not relevant to the choice of reflections for refinement. *R*-factors based on *F*^2^ are statistically about twice as large as those based on *F*, and *R*- factors based on ALL data will be even larger.

### Fractional atomic coordinates and isotropic or equivalent isotropic displacement parameters (Å^2^)

**Table:**

	*x*	*y*	*z*	*U*_iso_*/*U*_eq_	
O2	0.8613 (3)	0.64114 (19)	−0.0032 (2)	0.0693 (8)	
O3	0.4161 (3)	0.39290 (18)	0.2751 (2)	0.0755 (9)	
O4	0.4378 (2)	0.22073 (16)	0.2114 (2)	0.0594 (7)	
H4	0.3865	0.2064	0.2611	0.089*	
C1	0.7731 (3)	0.6172 (2)	0.0625 (3)	0.0418 (7)	
C2	0.7101 (3)	0.5018 (2)	0.0565 (2)	0.0358 (7)	
C3	0.6177 (3)	0.4682 (2)	0.1322 (2)	0.0363 (7)	
H3	0.5928	0.5181	0.1876	0.044*	
C4	0.5616 (3)	0.3611 (2)	0.1267 (2)	0.0364 (7)	
C5	0.5951 (3)	0.2889 (2)	0.0422 (3)	0.0481 (8)	
H5A	0.5568	0.2172	0.0373	0.058*	
C6	0.6849 (4)	0.3223 (3)	−0.0350 (3)	0.0555 (9)	
H6	0.7061	0.2734	−0.0926	0.067*	
C7	0.7434 (3)	0.4276 (2)	−0.0276 (3)	0.0483 (8)	
H7	0.8056	0.4492	−0.0791	0.058*	
C8	0.4642 (3)	0.3282 (2)	0.2117 (3)	0.0429 (7)	
O1	0.7347 (2)	0.68498 (17)	0.13536 (19)	0.0534 (6)	
O5	0.9635 (3)	0.83592 (18)	0.0383 (2)	0.0613 (7)	
H5	0.9340	0.7740	0.0166	0.092*	
N1	0.8850 (3)	0.8758 (2)	0.1251 (2)	0.0504 (7)	
H1	0.8150	0.8258	0.1335	0.060*	
C9	1.0396 (4)	0.7722 (4)	0.2759 (4)	0.0870 (13)	
H9A	0.9648	0.7224	0.2905	0.130*	
H9B	1.1032	0.7821	0.3456	0.130*	
H9C	1.0900	0.7410	0.2170	0.130*	
C10	0.9787 (4)	0.8832 (3)	0.2364 (3)	0.0697 (11)	
H10A	0.9251	0.9130	0.2951	0.084*	
H10B	1.0551	0.9349	0.2277	0.084*	
C11	0.7195 (5)	0.9731 (4)	−0.0227 (4)	0.0870 (13)	
H11A	0.7696	0.9443	−0.0830	0.131*	
H11B	0.6806	1.0452	−0.0450	0.131*	
H11C	0.6446	0.9226	−0.0101	0.131*	
C12	0.8190 (4)	0.9843 (3)	0.0861 (3)	0.0635 (10)	
H12A	0.8927	1.0374	0.0735	0.076*	
H12B	0.7680	1.0141	0.1465	0.076*	

### Atomic displacement parameters (Å^2^)

**Table:**

	*U*^11^	*U*^22^	*U*^33^	*U*^12^	*U*^13^	*U*^23^
O2	0.0955 (18)	0.0479 (14)	0.0763 (17)	−0.0257 (12)	0.0577 (15)	−0.0171 (12)
O3	0.106 (2)	0.0381 (13)	0.097 (2)	−0.0105 (12)	0.0701 (17)	−0.0130 (12)
O4	0.0767 (17)	0.0336 (12)	0.0770 (18)	−0.0074 (10)	0.0459 (13)	−0.0033 (10)
C1	0.0488 (17)	0.0335 (16)	0.0462 (17)	0.0001 (13)	0.0186 (14)	−0.0018 (13)
C2	0.0364 (14)	0.0309 (15)	0.0406 (16)	0.0006 (11)	0.0072 (12)	0.0016 (12)
C3	0.0409 (15)	0.0283 (15)	0.0414 (16)	0.0034 (11)	0.0123 (12)	−0.0022 (12)
C4	0.0360 (14)	0.0310 (15)	0.0432 (16)	0.0028 (11)	0.0085 (12)	0.0016 (12)
C5	0.0527 (18)	0.0322 (16)	0.063 (2)	−0.0059 (13)	0.0199 (15)	−0.0078 (14)
C6	0.072 (2)	0.0399 (18)	0.061 (2)	−0.0105 (15)	0.0341 (17)	−0.0197 (15)
C7	0.0584 (19)	0.0402 (17)	0.0521 (19)	−0.0041 (14)	0.0293 (15)	−0.0060 (14)
C8	0.0490 (17)	0.0308 (16)	0.0515 (18)	−0.0005 (13)	0.0168 (14)	−0.0026 (13)
O1	0.0594 (13)	0.0350 (12)	0.0728 (15)	−0.0094 (10)	0.0364 (12)	−0.0134 (10)
O5	0.0832 (17)	0.0478 (14)	0.0618 (15)	−0.0209 (12)	0.0448 (13)	−0.0137 (11)
N1	0.0623 (17)	0.0404 (15)	0.0540 (16)	−0.0215 (12)	0.0296 (13)	−0.0096 (12)
C9	0.077 (3)	0.115 (4)	0.069 (3)	0.006 (3)	0.011 (2)	0.003 (3)
C10	0.077 (3)	0.079 (3)	0.055 (2)	−0.024 (2)	0.0163 (19)	−0.0136 (19)
C11	0.099 (3)	0.068 (3)	0.093 (3)	−0.009 (2)	0.007 (3)	0.007 (2)
C12	0.083 (2)	0.0390 (19)	0.075 (2)	−0.0208 (17)	0.035 (2)	−0.0081 (16)

### Geometric parameters (Å, °)

**Table:**

O2—C1	1.238 (3)	O5—N1	1.412 (3)
O3—C8	1.198 (3)	O5—H5	0.8200
O4—C8	1.307 (4)	N1—C10	1.486 (5)
O4—H4	0.8200	N1—C12	1.487 (5)
C1—O1	1.259 (3)	N1—H1	0.9100
C1—C2	1.502 (4)	C9—C10	1.496 (6)
C2—C3	1.380 (4)	C9—H9A	0.9600
C2—C7	1.386 (4)	C9—H9B	0.9600
C3—C4	1.384 (4)	C9—H9C	0.9600
C3—H3	0.9300	C10—H10A	0.9700
C4—C5	1.375 (4)	C10—H10B	0.9700
C4—C8	1.491 (4)	C11—C12	1.495 (6)
C5—C6	1.374 (4)	C11—H11A	0.9600
C5—H5A	0.9300	C11—H11B	0.9600
C6—C7	1.374 (4)	C11—H11C	0.9600
C6—H6	0.9300	C12—H12A	0.9700
C7—H7	0.9300	C12—H12B	0.9700
			
C8—O4—H4	109.5	C10—N1—C12	113.8 (3)
O2—C1—O1	123.0 (3)	O5—N1—H1	108.4
O2—C1—C2	118.7 (2)	C10—N1—H1	108.4
O1—C1—C2	118.3 (2)	C12—N1—H1	108.4
C3—C2—C7	119.1 (3)	C10—C9—H9A	109.5
C3—C2—C1	121.3 (2)	C10—C9—H9B	109.5
C7—C2—C1	119.6 (2)	H9A—C9—H9B	109.5
C2—C3—C4	120.8 (3)	C10—C9—H9C	109.5
C2—C3—H3	119.6	H9A—C9—H9C	109.5
C4—C3—H3	119.6	H9B—C9—H9C	109.5
C5—C4—C3	119.3 (3)	N1—C10—C9	112.7 (3)
C5—C4—C8	121.9 (3)	N1—C10—H10A	109.0
C3—C4—C8	118.8 (2)	C9—C10—H10A	109.0
C6—C5—C4	120.3 (3)	N1—C10—H10B	109.0
C6—C5—H5A	119.8	C9—C10—H10B	109.0
C4—C5—H5A	119.8	H10A—C10—H10B	107.8
C7—C6—C5	120.3 (3)	C12—C11—H11A	109.5
C7—C6—H6	119.8	C12—C11—H11B	109.5
C5—C6—H6	119.8	H11A—C11—H11B	109.5
C6—C7—C2	120.2 (3)	C12—C11—H11C	109.5
C6—C7—H7	119.9	H11A—C11—H11C	109.5
C2—C7—H7	119.9	H11B—C11—H11C	109.5
O3—C8—O4	123.1 (3)	N1—C12—C11	112.5 (3)
O3—C8—C4	123.8 (3)	N1—C12—H12A	109.1
O4—C8—C4	113.1 (2)	C11—C12—H12A	109.1
N1—O5—H5	109.5	N1—C12—H12B	109.1
O5—N1—C10	109.4 (3)	C11—C12—H12B	109.1
O5—N1—C12	108.4 (2)	H12A—C12—H12B	107.8
			
O2—C1—C2—C3	176.0 (3)	C5—C6—C7—C2	−1.3 (5)
O1—C1—C2—C3	−3.1 (4)	C3—C2—C7—C6	0.1 (5)
O2—C1—C2—C7	−4.8 (4)	C1—C2—C7—C6	−179.1 (3)
O1—C1—C2—C7	176.0 (3)	C5—C4—C8—O3	−168.5 (3)
C7—C2—C3—C4	1.6 (4)	C3—C4—C8—O3	10.4 (4)
C1—C2—C3—C4	−179.2 (3)	C5—C4—C8—O4	12.4 (4)
C2—C3—C4—C5	−2.1 (4)	C3—C4—C8—O4	−168.7 (3)
C2—C3—C4—C8	178.9 (2)	O5—N1—C10—C9	−60.7 (4)
C3—C4—C5—C6	0.9 (4)	C12—N1—C10—C9	177.9 (3)
C8—C4—C5—C6	179.8 (3)	O5—N1—C12—C11	61.2 (4)
C4—C5—C6—C7	0.8 (5)	C10—N1—C12—C11	−176.9 (3)

### Hydrogen-bond geometry (Å, °)

**Table:**

*D*—H···*A*	*D*—H	H···*A*	*D*···*A*	*D*—H···*A*
N1—H1···O1	0.91	1.85	2.702 (4)	155
O5—H5···O2	0.82	1.74	2.545 (4)	169
O4—H4···O1^i^	0.82	1.79	2.606 (4)	175
C10—H10A···O2^ii^	0.97	2.58	3.373 (5)	139
C12—H12A···O5^iii^	0.97	2.51	3.426 (5)	157
C12—H12B···O3^iv^	0.97	2.53	3.117 (5)	119

Symmetry codes: (i) −*x*+1, *y*−1/2, −*z*+1/2; (ii) *x*, −*y*+3/2, *z*+1/2; (iii) −*x*+2, −*y*+2, −*z*; (iv) −*x*+1, *y*+1/2, −*z*+1/2.
